# Electric Shock Sensation and Recurrent Falls as a Side Effect of Clozapine Therapy: A Case Report

**DOI:** 10.7759/cureus.61143

**Published:** 2024-05-26

**Authors:** Beenish Mujahid, Lubna Lutfi, Abdelaziz Alhassan

**Affiliations:** 1 Department of Psychiatry, Al Amal Hospital, Dubai, ARE; 2 Department of Psychiatry, Al Amal Psychiatric Hospital, Dubai, ARE

**Keywords:** antipsychotic medication, aripiprazole, schizoaffective disorder, treatment-resistant schizophrenia, clozapine

## Abstract

Clozapine is an atypical antipsychotic that acts by blocking mainly dopamine 4 receptors. It is usually prescribed for treatment-resistant schizophrenia as well as treatment-resistant bipolar disorder. Clozapine has a wide profile of side effects that result from blocking different receptors all over the body. A 42-year-old Middle Eastern female is known to have suffered from schizoaffective disorder for many years and had frequent relapses despite compliance with treatment. She was commenced on Clozapine; the patient started complaining of an electric shock sensation throughout her body that resulted in recurrent falls with bilateral leg fractures. She was started on sodium valproate to exclude the possibility of seizure activity but the electric shock sensation did not subside. The decision was made to switch her to aripiprazole and gradually taper down and stop Clozapine which improved her symptoms. Careful monitoring of patients who receive Clozapine is recommended especially during the tapering phase due to the risky adverse events it can bring about. It is essential to understand the side effects in order to tackle them as soon as they arise.

## Introduction

Clozapine is an atypical antipsychotic that acts by blocking dopamine 4 receptors mainly [1] as well as dopamine 2 and serotonin 2A receptors. It is usually prescribed for treatment-resistant schizophrenia as well as treatment-resistant bipolar disorder. Clozapine has a wide profile of side effects that result from blocking different receptors including but not limited to alpha-adrenergic receptors, muscarinic receptors, and histamine receptors. Life-threatening side effects of Clozapine use include agranulocytosis, myocarditis, seizures, and paralytic ileus [2]. Our case reports the feeling of electric shock all over the body resulting in recurrent falls while on Clozapine monotherapy for treatment-resistant schizoaffective disorder.

## Case presentation

A 42-year-old Middle Eastern female, single and unemployed was known to have suffered from schizoaffective disorder for many years. The patient had multiple previous admissions to psychiatric facilities due to frequent relapses despite compliance with medications and regular follow-up appointments as an outpatient, and those episodes of relapse would start just a few months after getting discharged from the hospital. The relapsing symptoms often included persecutory delusions, persistent auditory hallucinations that are commanding in nature, and aggression toward her family members.

During hospitalization, medications would be adjusted and optimized until efficacy was noted, she was always managed with a combination of two antipsychotics (typical or atypical) and one antiepileptic mood stabilizer. Electroconvulsive therapy was not tried with the patient.

In October 2022, the patient was hospitalized again during which all her past medications were stopped and she was commenced on Clozapine, which was tapered up to 500 mg daily to tackle her psychotic symptoms, and then discharged after she was stable on this dosage.

One week after discharge, the patient started complaining of an electric shock sensation throughout her body for the first time ever, she reports that it happened more than one time during the day and it resulted in her falling. Due to this side effect, Clozapine was gradually reduced to 300 mg daily in divided doses.

In March 2023, the patient’s family brought her into the hospital to be under observation after the first fall she experienced; her psychiatric risk assessment was negative. She, however, did report the same auditory hallucinations.

**Figure 1:**
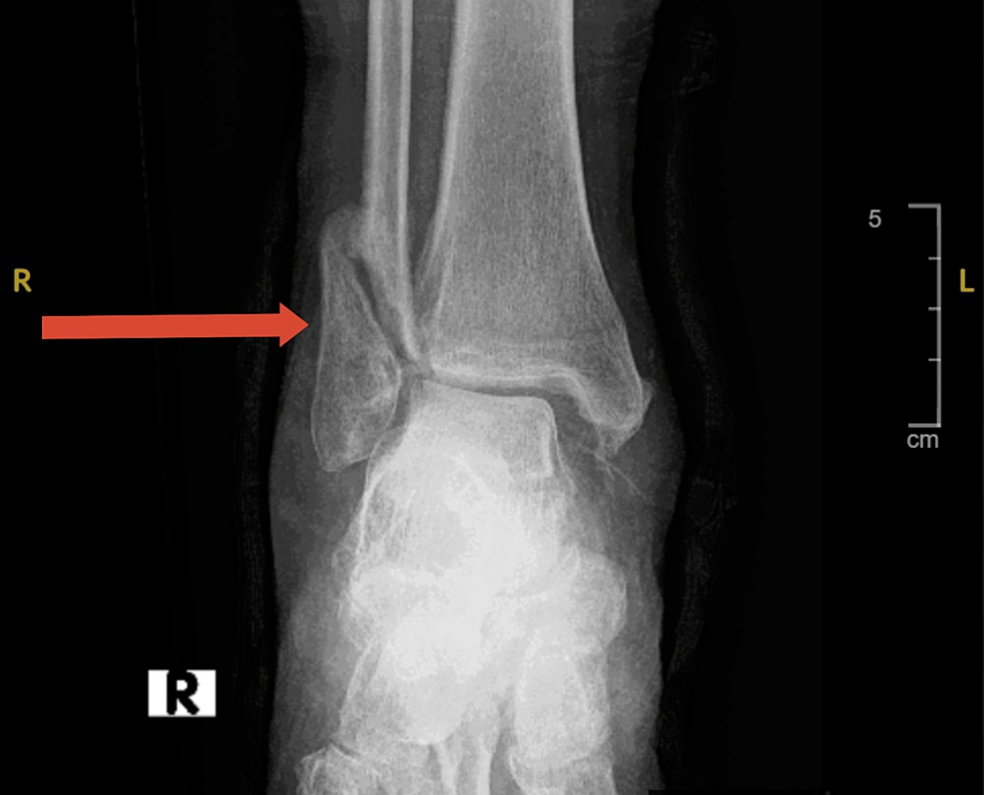
Right leg fracture Fracture distal end shaft fibula.

Upon arrival to the ward, she was seen sitting in a wheelchair, however, she was able to bear weight on her right leg and had a stable gait. When interviewed, the patient described the feeling of an electric shock that occurs at random throughout her body and causes her to lose balance and fall. A right leg X-ray revealed a fibular fracture (Figure 1) and an orthopedics referral was carried out. Their advice was to conservatively manage her with a cast for a few weeks then to come back for follow-up as an outpatient.

The patient stayed in the hospital for 2 weeks and continued on the same dose of Clozapine. During her time she was still complaining of the same electric shock sensation in her body, mainly in both of her lower limbs. As the treating psychiatrists suspected myoclonus or seizures as a side effect of Clozapine therapy, she was started on sodium valproate to exclude the possibility of seizure activity.

**Figure 2:**
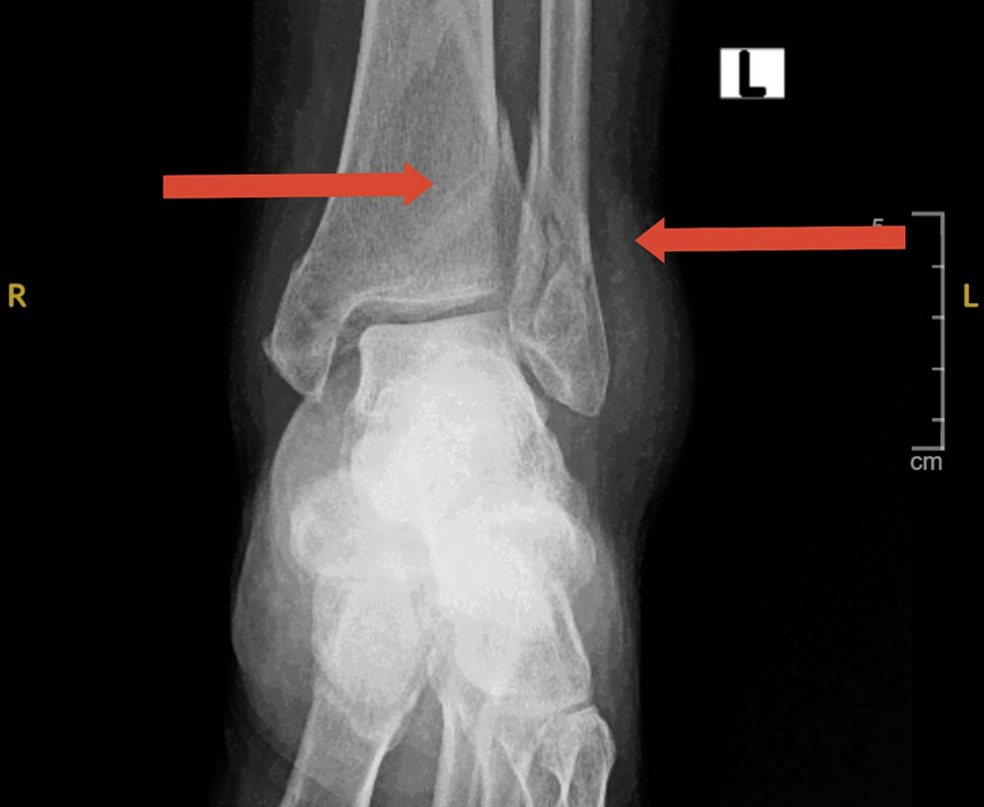
Left leg fracture Comminuted fracture lower tibia and fibula.

The patient left with her family for a few days and when she returned they reported one more fall. Upon interviewing the patient, she was still complaining of the same electric sensation that results in her losing balance and falling, she denied any jerky movements, incontinence, uprolling of the eyes, or frothing from the mouth, she also denied loss of consciousness but did complain of dizziness. Orthopedics referral was done and lower leg x-rays revealed bilateral tibial shaft fractures (Figure 2) in addition to the previous fibular shaft fracture. This time they recommended surgical management.

The treating psychiatrists decided to stop sodium valproate as this trial helped exclude the possibility of seizures, and to slowly taper down Clozapine due to the serious side effects the patient experienced and as requested by her family. She was started on oral aripiprazole and monthly long-acting aripiprazole injections.

The patient came back for a monthly follow-up in the outpatient department, she and her family reported marked improvement with residual auditory hallucinations and muttering to self only and no other psychotic symptoms 3 months after being on aripiprazole monotherapy.

## Discussion

Clozapine has historically been effective as long-term therapy in treatment-resistant schizophrenia, psychotic bipolar disorder, and schizoaffective disorder. It is indicated in treatment-resistant cases and cases of psychosis with a high risk of suicide. Treatment resistance is described as the persistence of positive symptoms, delusions, and hallucinations after the trial of two or more antipsychotic therapies [3]. Our patient had trials of multiple antipsychotic medications through the years as mentioned earlier, some in combinations, and was found to be difficult to treat as she persistently experienced residual symptoms despite adherence and change in management plans which is why Clozapine was considered as monotherapy.

Commencing Clozapine therapy should come with multiple considerations and careful titration due to the numerous possible adverse drug reactions (ADRs). Common side effects include orthostatic hypotension and sexual dysfunction due α adrenergic receptor block. It is worth noting that orthostatic hypotension was considered as an ADR resulting in falls in our case; however, she was monitored for that and observed by the treating team and it was not the cause of her falls. Constipation, tachycardia, and urinary retention can result due to M1 anti-muscarinic receptor block. In addition to, sedation and constipation due to H1 histamine receptors block. Nausea, dizziness, and sialorrhea are also commonly experienced [4].

Clozapine can also result in more serious and life-threatening side effects. Agranulocytosis affects almost 1% of patients on Clozapine and is detected by regularly scheduled complete blood counts [5]. Myocarditis and cardiomyopathy are rare side effects that affect 0.006-0.007% of individuals in the first 4 weeks of treatment according to a meta-analysis published in 2020 [6] for which electrocardiogram and baseline cardiac enzymes are advised to be monitored. Metabolic syndrome comprising of increased body mass index, increased lipid levels in the blood, and type 2 diabetes also results from the drug’s high affinity to serotonin 5-HT2C receptors and can be managed by lifestyle modification and adjuvant therapy using metformin, orlistat, and aripiprazole as evident in a meta-analysis published in 2016 [4].

Recurrent falls and an electric shock sensation have been described in the literature as a form of myoclonus that occurs as a side effect of Clozapine therapy and possibly turns into a full-blown generalized seizure later on [7-10].

The incidence of Clozapine-induced myoclonus is not very clear, possibly due to variations in presentation and underreporting. A review of the literature estimated the incidence to be 0.9% to 12.5%. Myoclonus can present as jerky movements in the limb and trunk, interruption of motor activity, orofacial jerks, or unexplained falls [7].

Furthermore, seizures are a relatively common and potentially life-threatening adverse effect of Clozapine. In fact, Clozapine may lower the seizure threshold not only in individuals at risk of epilepsy but also in apparently healthy subjects. The incidence rate varies in different studies. A paper published in 2018 estimated the risk of developing seizures at roughly 1-6% and higher doses of Clozapine (⩾600 mg/day) were associated with higher risk [4].

A literature review estimated an incidence rate of 4% up to 20%. The most common types of seizures as described in the same paper are generalized tonic-clonic or generalized myoclonic seizures [11].

Another study estimated a frequency of 1.3% and revealed that patients who had a history of seizures or epilepsy were more prone to developing seizures [12]. One more paper reported that 20% of patients developed seizures while 74% had EEG abnormalities, generalized tonic-clonic seizures were the most common form [13].

Our patient described her symptoms in a very similar manner; an electric shock sensation that occurs all over her lower limbs that she is unable to control and results in her losing balance and falling; however, there were no frank seizure activities noted or observed by the team. Though an EEG was not conducted, a trial of sodium valproate was given with the rationale that if it was seizure activity then it would help reduce it. Management of Clozapine-induced seizures of tonic-clonic, myoclonic, or atonic natures can be summarized in (1) lowering the dose (2) starting antiepileptic medication, namely sodium valproate (3) and finally switching to another antipsychotic [7,14].

It is important to also acknowledge other rare side effects that have been addressed in the literature because of Clozapine treatment. Drug reaction with eosinophilia and systemic symptoms (DRESS syndrome), which is a severe and multi-organ hypersensitivity reaction, occurred in 12 reported cases within 1 month of starting Clozapine therapy as per a systemic review published in 2021 [15]. Pseudopheochromocytoma manifested as hypertension and detected by urinary catecholamines was reported in four cases of patients receiving Clozapine after excluding other organic causes [16]. Periorbital edema [17], necrotizing colitis [18], and renal failure [19] have also been reported as independent case reports. A meta-analysis also found a 2.09-2.7% incidence rate of appendicitis when compared to patients on Clozapine vs other antipsychotic agents [20].

The significance of our case lies in the fact that not many cases of a similar nature were reported before. We have excluded organic causes like orthostatic hypotension by monitoring and examining that directly and any brain abnormality by conducting a brain CT scan when she first presented to us. We excluded the possibility of seizure activity when her symptoms did not improve with sodium valproate; however, we did not carry out an EEG and relied on the patient’s own description of her symptoms. It is worth noting that her symptoms subsided after stopping Clozapine. However, it is highly recommended to perform an EEG to properly identify any seizure activity to solidify suspicions, though it is also of utmost value to listen to the patient’s complaint and adjust her treatment as is best tolerated by her. It is also recommended to monitor the Clozapine level in the blood during the tapering phase and titrate the dose accordingly. Finally, Clozapine alone does not result in extrapyramidal side effects that can present as movement abnormalities, so it is recommended to examine all abnormal movements that occur during the course of treatment.

## Conclusions

Our patient experienced an electric shock sensation all over her body that caused multiple falls resulting in bilateral lower limb fractures for which she required orthopedic management. Clozapine therapy has certainly been an effective agent in the treatment of refractory or treatment-resistant cases, but it needs to be approached with caution. Careful monitoring of patients who receive it is recommended, especially during the titration phase due to the risky adverse events it can bring about. It is crucial to understand its side effects in order to tackle them as early as they arise, and it is also essential to report the less common or rare adverse events. This helps achieve the ultimate goal in medical management which is to reduce symptoms all while improving the patient’s lifestyle.
